# The Role of Minority Stress and Social Resources in the Healthcare Utilization of Aging LGBT Adults

**DOI:** 10.1093/geroni/igab046.660

**Published:** 2021-12-17

**Authors:** Krystal Kittle, Kathrin Boerner, Kyungmin Kim

**Affiliations:** 1 University of Nevada, Las Vegas, Las Vegas, Nevada, United States; 2 University of Massachusetts Boston, Boston, Massachusetts, United States; 3 Seoul National University, Seoul, Seoul-t'ukpyolsi, Republic of Korea

## Abstract

Research suggests that minority stress can influence the healthcare utilization of aging LGBT adults, and that social resources can buffer the effect of stress on healthcare utilization. Using data from Aging with Pride: National Health, Aging, and Sexuality/Gender Study (N = 2,560), multiple logistic regression assessed the associations between minority stress (i.e., internalized stigma and LGBT identity disclosure) and healthcare utilization (i.e., health screenings, emergency room use, routine checkups, and regular provider). We also examined the moderating effect of social resources, including social network size, social support, and LGBT community belonging, in these associations. Internalized stigma was negatively associated with having a routine checkup in the previous year (OR = 0.82, p = .038). Disclosure was positively associated with having a health screening within the past 3 years (OR = 1.52, p = .000) and having a regular provider (OR = 1.33, p = .021). Further, we found that social support moderated the association between disclosure and health screenings (OR = 1.52, p < .001); thus, having higher levels of social support and disclosure in tandem increased the likelihood of getting a health screening in the last three years. Health and human service professionals should provide information about internalized stigma and LGBT identity disclosure to educate their clients about the ways in which these minority stressors can impact their healthcare experiences. Providers should assess the social support of their aging LGBT clients and inform them about the added benefit that social support can have in their healthcare experiences.

